# The prevalence of small intestinal bacterial overgrowth in diabetes mellitus: a systematic review and meta-analysis

**DOI:** 10.18632/aging.203854

**Published:** 2022-01-27

**Authors:** Xin Feng, Xiao-Qing Li

**Affiliations:** 1 Department of Gastroenterology, The People’s Hospital of Yubei District of Chongqing City, Chongqing 401120, China; 2 Department of Gastroenterology, Chongqing University Cancer Hospital, Shapingba, Chongqing 400030, China

**Keywords:** small intestinal bacterial overgrowth, diabetes mellitus

## Abstract

Objective: We conducted this systematic review and meta-analysis to estimate the prevalence of SIBO in diabetic patients and to determine the association between SIBO and diabetes.

Methods: A comprehensive literature search of the PubMed, Cochrane Library and EMBASE databases from inception to June 2021 was conducted for studies correlating SIBO with diabetes. Studies were screened, and relevant data were extracted and analysed. The pooled prevalence of SIBO among diabetic patients and the odds ratio of SIBO among diabetic patients compared with controls were calculated.

Results: Fourteen studies including 1417 diabetic patients and 649 controls met the inclusion criteria. The pooled prevalence of SIBO in diabetes was 29% (95% CI 20–39%). The odds ratio of SIBO in diabetic patients was 2.91 (95% CI 0.82–10.32, p=0.1) compared with controls. Subgroup analyses showed that the prevalence of SIBO in diabetes was higher in studies using jejunal aspirate culture for diagnosis (39%, 95% CI 12–66%) than in those using the lactulose breath test (31%, 95% CI 18–43%) or glucose breath test (29%, 95% CI 14–43%). The prevalence of SIBO in diabetes was higher in studies conducted in Western countries (35%, 95% CI 21–49%) than in those conducted in Eastern countries (24%, 95% CI 14–34%), and the prevalence of SIBO in type 1 diabetes (25%, 95% CI 14%–36%) was not significantly different from that in type 2 diabetes (30%, 95% CI 13%–47%).

Conclusions: Twenty-nine percent of diabetic patients tested positive for SIBO, and the risk of SIBO in diabetic patients was 2.91 times higher than that in patients without diabetes. Diabetes could be a predisposing factor for the development of SIBO, especially among patients diagnosed by jejunal aspirate culture or those in Western populations.

## INTRODUCTION

Small intestinal bacterial overgrowth (SIBO) is defined as the presence of excessive numbers of bacteria and/or abnormal types of bacteria in the small bowel, causing gastrointestinal symptoms that include malnutrition, diarrhoea and abdominal distension [1]. The normal intestinal microbial balance is maintained by many important mechanisms that include gastric acid secretion, anatomical integrity of the digestive tract, propulsive peristaltic activity, and secretory IgA immunoglobulins [2]. Failure of these mechanisms can be responsible for the development of SIBO. The gold standard for diagnosing SIBO is jejunal aspirate culture (JAC). Alternatively, the breath test, a widely used method for diagnosing SIBO, has the advantages of being simple, non-invasive and easily acceptable. Recent studies have shown that SIBO is closely associated with various diseases, including Crohn's disease [3], irritable bowel syndrome [4], functional dyspepsia [5], hepatic encephalopathy [6], and non-alcoholic fatty liver disease [7].

Diabetes mellitus (DM) is a serious and growing global public health burden [8]. DM was estimated to affect at least 382 million people worldwide in 2013, and this number will rise to 592 million by the year 2035 [9]. DM is a metabolic disease characterized by hyperglycaemia, which can cause multiple-organ damage. Gastrointestinal complications are common among patients with DM [10, 11]. Diabetes patients have been reported to exhibit increased risks of SIBO [12], but several studies have reported inconsistent results [13, 14]. Therefore, we conducted a systematic review and meta-analysis to investigate the relationship between DM and the risk of SIBO.

## MATERIALS AND METHODS

This meta-analysis was conducted in accordance with the Preferred Reporting Items for Systematic reviews and Meta-Analyses (PRISMA) recommendations [15].

### Search strategy

We searched the PubMed, Cochrane Library and EMBASE databases from their inception to June 2021 using the following search terms: (diabetes mellitus OR diabetes OR diabetic OR T1DM OR T2DM) AND (small intestinal bacterial overgrowth OR small intestine bacterial overgrowth OR SIBO OR small bowel bacterial overgrowth OR breath test OR SBBO). The literature search had no language restrictions. We also screened the reference lists of the included studies and relevant reviews to identify all eligible articles. Two reviewers (X. Feng and XQ. Li) independently performed the literature search.

### Study selection

Articles were eligible if they met the following criteria: (a) cohort studies, case–control studies or cross-sectional studies investigating the relationship between SIBO and DM; (b) subjects > 18 years old; (c) studies that recruited subjects meeting the DM diagnostic criteria; (d) valid methods used to assess SIBO, including the lactulose breath test (LBT), glucose breath test (GBT) or JAC; and (e) studies available in a full-text format. We excluded articles such as case reports, review articles, letters and those reporting animal research. In addition, we excluded studies that provided duplicate data. We did not determine the cut-off values for a positive test as long as the positive criteria were clarified. When a study used more than one test to diagnose SIBO, we extracted data from each method separately.

### Data extraction and quality assessment

Two reviewers (X. Feng and XQ. Li) independently extracted the following data from the included studies: first author’s surname, year of publication, origin of study, study design, diagnostic test for SIBO, SIBO diagnostic criteria, prevalence of SIBO in DM, type of diabetes (type 1, type 2 or both), average age, sex, and course of diabetes. Any discrepancies between the two reviewers were resolved by a third author (Z. Jiang). Two reviewers (X. Feng and XQ. Li) independently evaluated the quality of the cohort studies or case–control studies with the Newcastle–Ottawa Scale (NOS) [16] and assessed the quality of the cross-sectional studies with the modified Newcastle–Ottawa Scale [17]. Studies with a score ≥7 were considered to be of high quality, while those with a score < 7 were considered to be of low quality.

### Statistical analysis

The pooled prevalence of SIBO in diabetic patients was calculated. Subgroup analyses were conducted by SIBO diagnostic tests (LBT vs. GBT vs. JAC), geographic areas (Western countries vs. Eastern countries) and type of diabetes (type 1 [T1DM] vs. type 2 [T2DM]). For cohort studies or case–control studies, the number of patients with SIBO in the case group and control group was calculated separately, and the odds ratios (ORs) and 95% confidence intervals (CIs) for the prevalence of SIBO in diabetic patients and their respective controls were then calculated. P values <0.05 were considered statistically significant. We used the Cochran Q statistic and I^2^ statistic to assess heterogeneity. An I^2^ value >50% or a P value <0.10 indicated statistically significant heterogeneity. The random-effects model was used with statistically significant heterogeneity; otherwise, the fixed-effects model was used. Furthermore, we used Egger’s test and funnel plot and a risk of bias graph to assess any potential publication bias. P >0.05 in Egger’s test was considered to indicate no publication bias. We also performed sensitivity analyses by omitting one study in turn, which investigated the effect of an individual study on the overall prevalence of SIBO. All statistical analyses were performed using R 3.5.3 or RevMan 5.3.

### Ethics approval and consent to participate

The manuscript has been read and approved by all of the authors, and the requirements for authorship, as stated earlier in this document, have been met.

### Availability of data and material

The data and material are available from the corresponding author upon request.

## RESULTS

**Figure 1:**
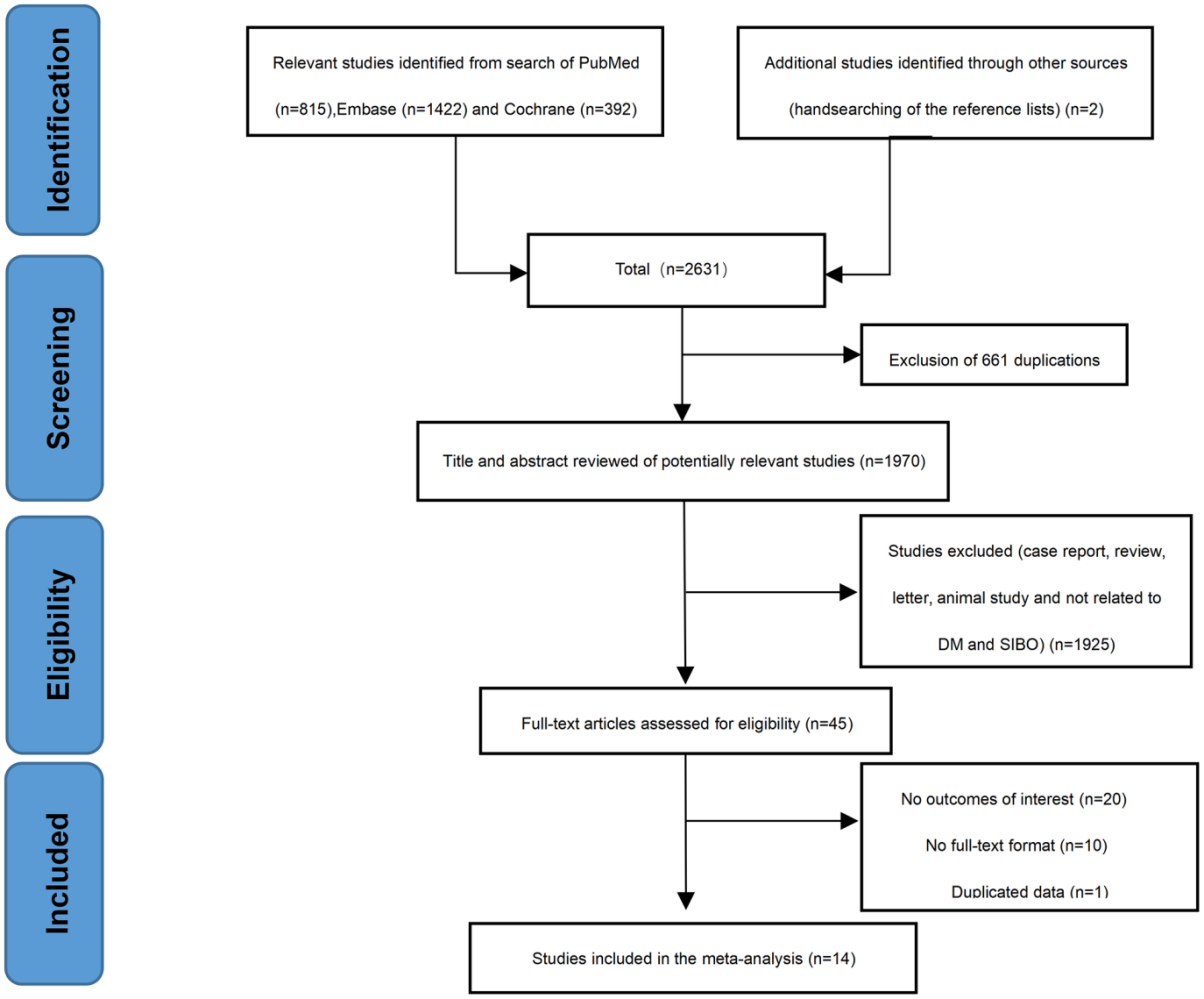
Flow chart of the selection process of articles.

The initial literature search revealed 2629 potentially relevant studies (815 from PubMed, 1422 from EMBASE and 392 from Cochrane Library). Two studies were added by hand-searching the references from the included studies. We excluded 661 duplicates. Subsequently, we excluded 1925 studies that did not meet our inclusion criteria, which resulted in a full-text review of 45 studies. Twenty articles that did not report outcomes of interest were excluded, and 10 articles were excluded because they were not full-text articles. One article was excluded because it duplicated data from another. Finally, 14 studies [12–14, 18–28] (9 cohort studies and 5 cross-sectional studies), including 2066 participants (1417 diabetics and 649 controls), were included in this meta-analysis (Figure 1). Since two different diagnostic tests for SIBO were performed with different results in one study [28], we separately calculated the prevalence of SIBO in two different studies. The characteristics and quality evaluation of the included studies are shown in Supplementary Table 1. All 14 articles were of high quality.

### Prevalence of SIBO in diabetic patients

**Figure 2:**
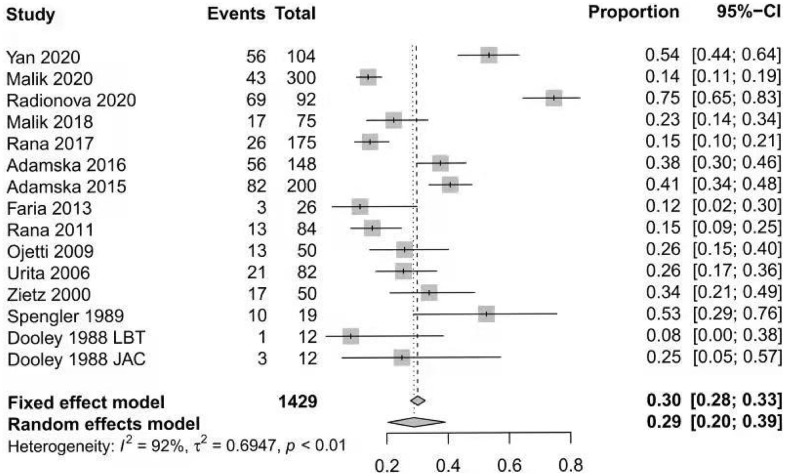
Forest plot of the pooled prevalence of SIBO in DM.

**Figure 3:**
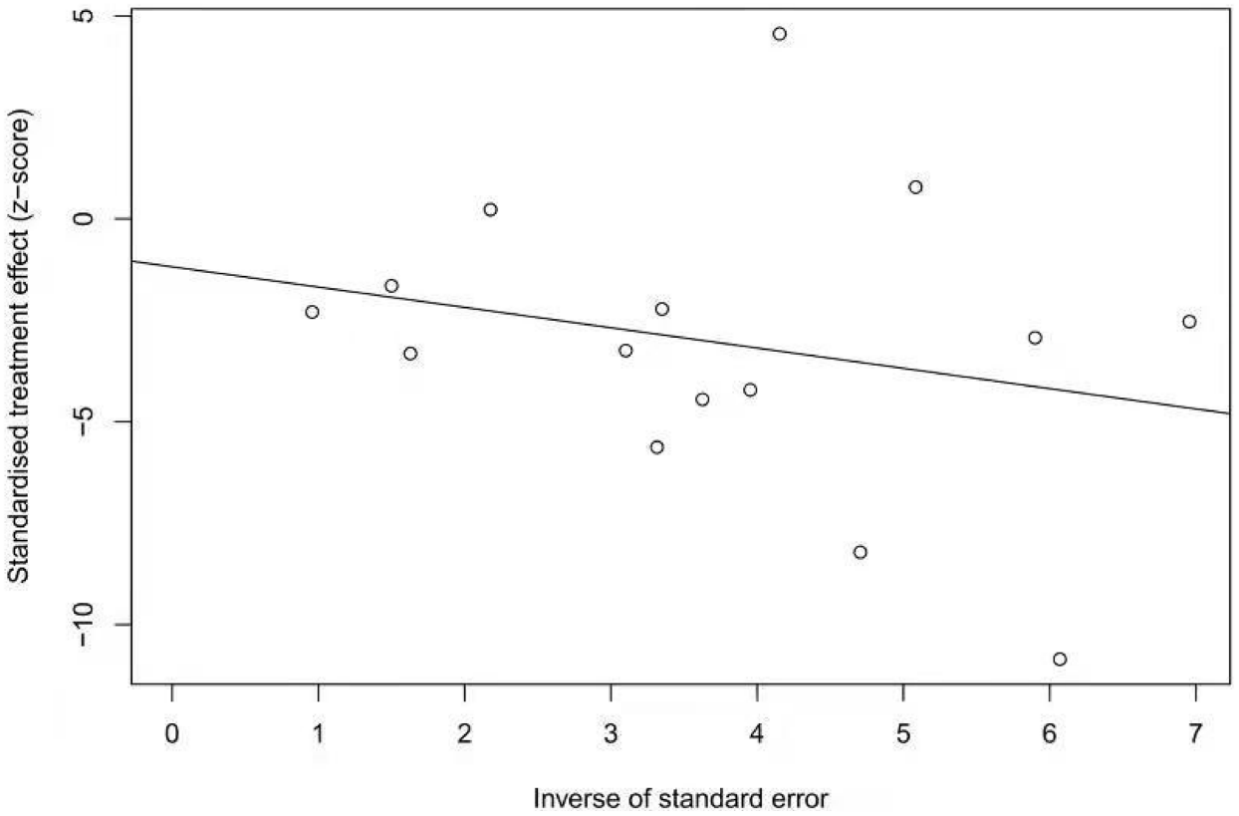
Egger test showing the publication bias of the pooled prevalence of SIBO in DM (p=0.6137).

**Figure 4:**
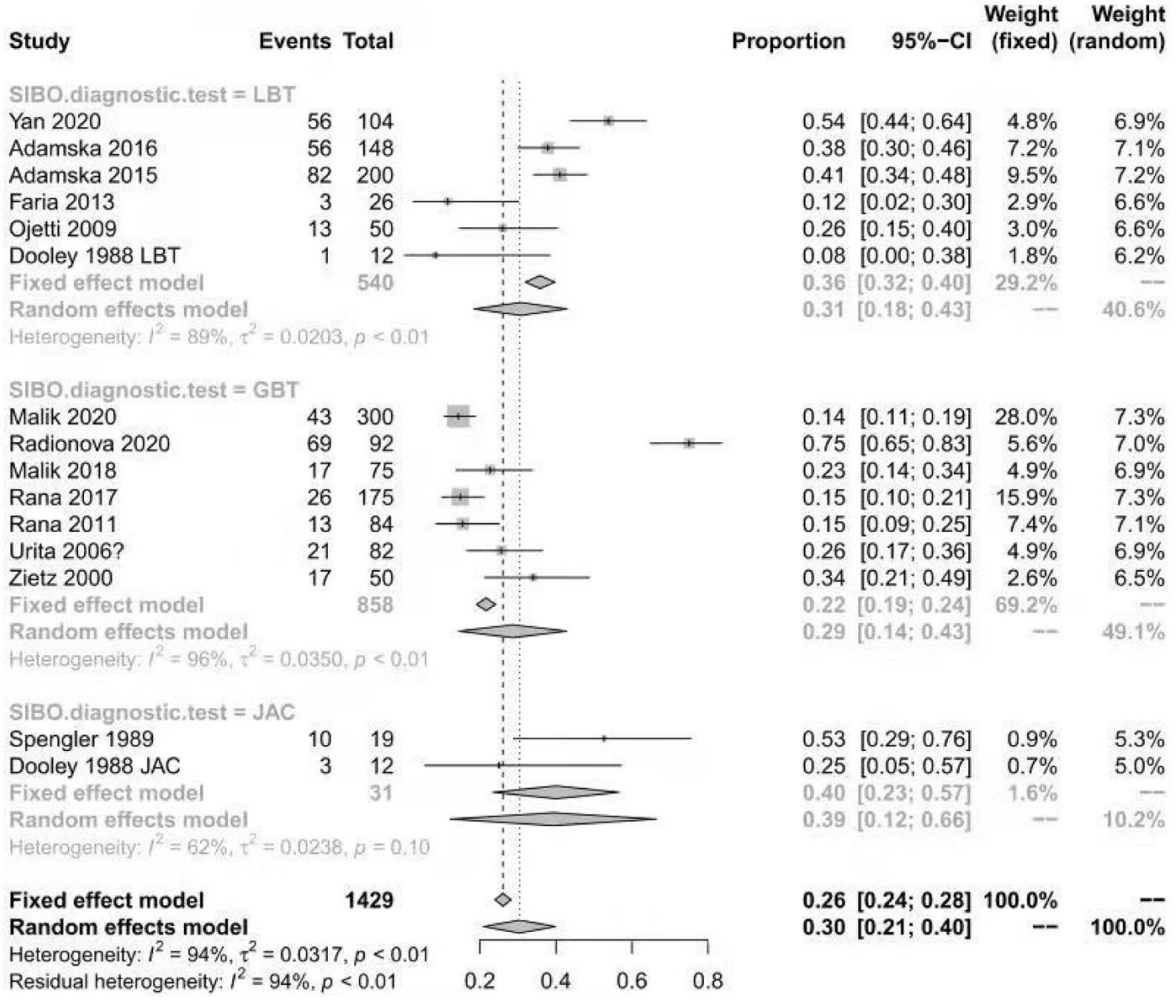
Forest plot of the prevalence of SIBO in DM based on the SIBO diagnostic test.

**Figure 5:**
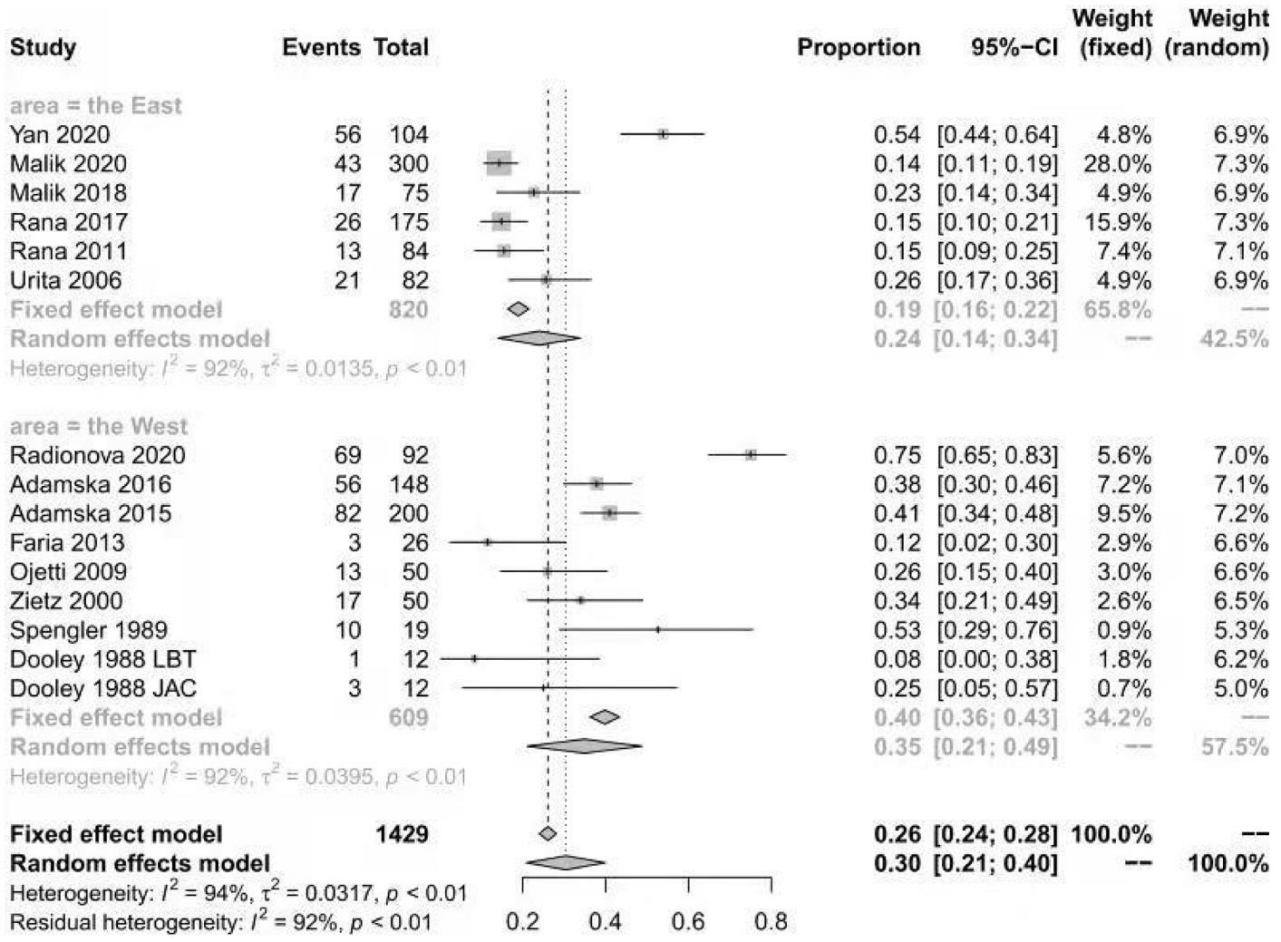
Forest plot of the prevalence of SIBO in DM based on geographic areas.

**Figure 6:**
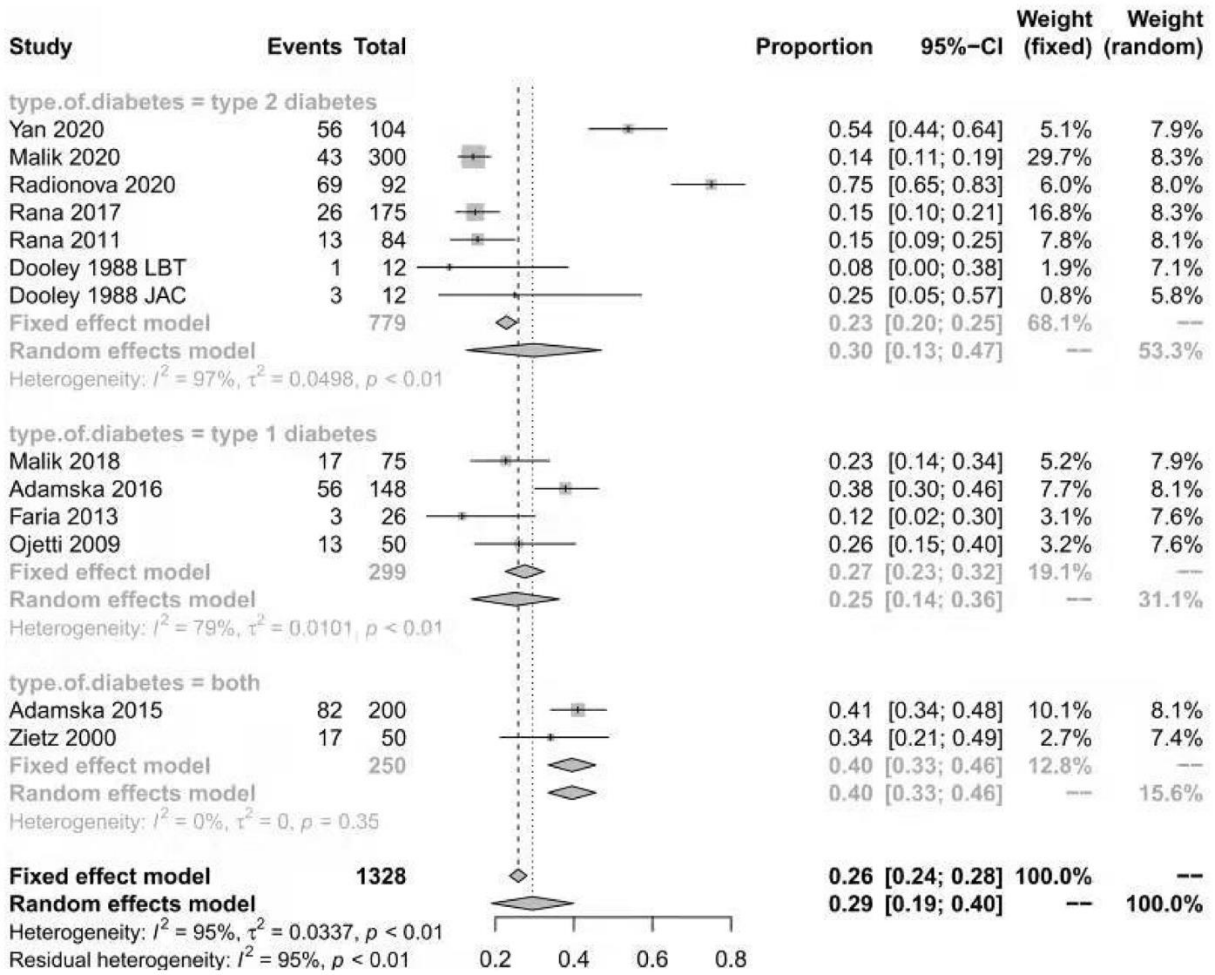
Forest plot of the prevalence of SIBO in DM based on type of diabetes.

The prevalence of SIBO in diabetic patients was reported in all included studies [12–14, 18–28] and ranged from 8% to 75%. The pooled prevalence of SIBO was 29% (95% CI 20%–39%), with considerable heterogeneity (I^2^=92%) (Figure 2). We used a random-effects model. The results of Egger’s test showed that there was no publication bias (P>0.05) (Figure 3). The studies were subgrouped based on the SIBO diagnostic test used. The prevalence of SIBO was 31% (95% CI 18%–43%) in six studies [13, 14, 18, 22, 24, 28] using the LBT and 29% (95% CI 14%–43%) in seven studies [12, 19–21, 23, 25, 26] using the GBT. Two studies [27, 28] using JAC showed a prevalence of 39% (95% CI 12%–66%) (Figure 4). When subgrouped by geographic area, the prevalence of SIBO was 35% (95% CI 21%–49%) in eight studies [13, 14, 19, 22, 24, 26–28] from Western countries and 24% (95% CI 14%–34%) in six studies [12, 18, 20, 21, 23, 25] from Eastern countries (Figure 5). Furthermore, in subgroup analysis based on the type of diabetes, the prevalence of SIBO in type 2 diabetes (30%, 95% CI 13%–47%) [12, 18, 19, 21, 23, 28] was similar to the prevalence in type 1 diabetes (25%, 95% CI 14%–36%) [13, 20, 22, 24]. The prevalence of SIBO in studies including both type 1 and type 2 diabetes [14, 26] was 40% (95% CI 33%–46%) (Figure 6).

### SIBO in diabetic patients compared with controls

**Figure 7:**
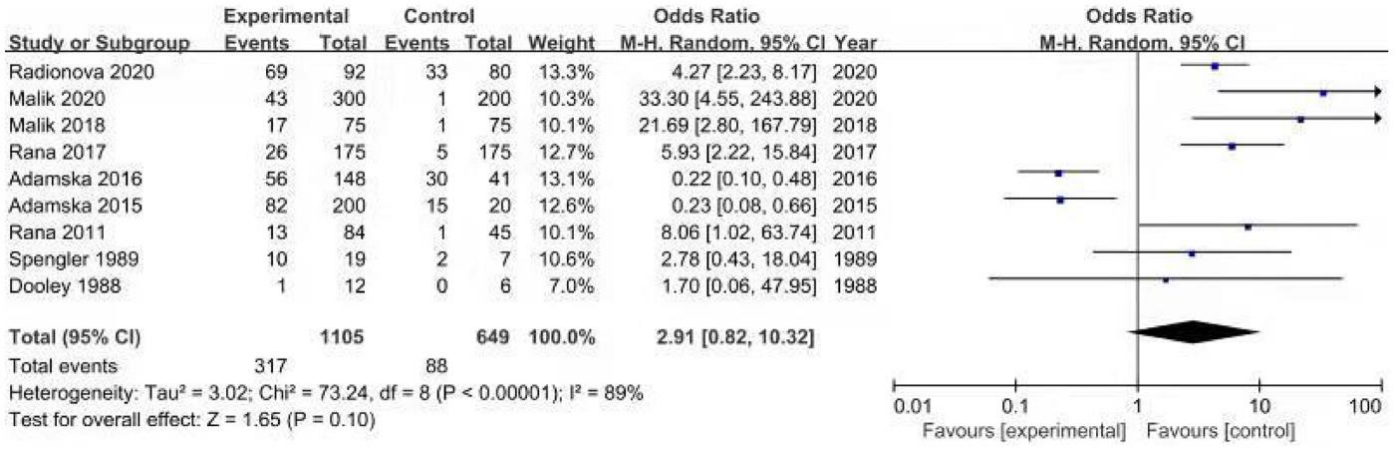
Forest plot of odds ratios of SIBO in diabetes patients compared with controls.

**Figure 8:**
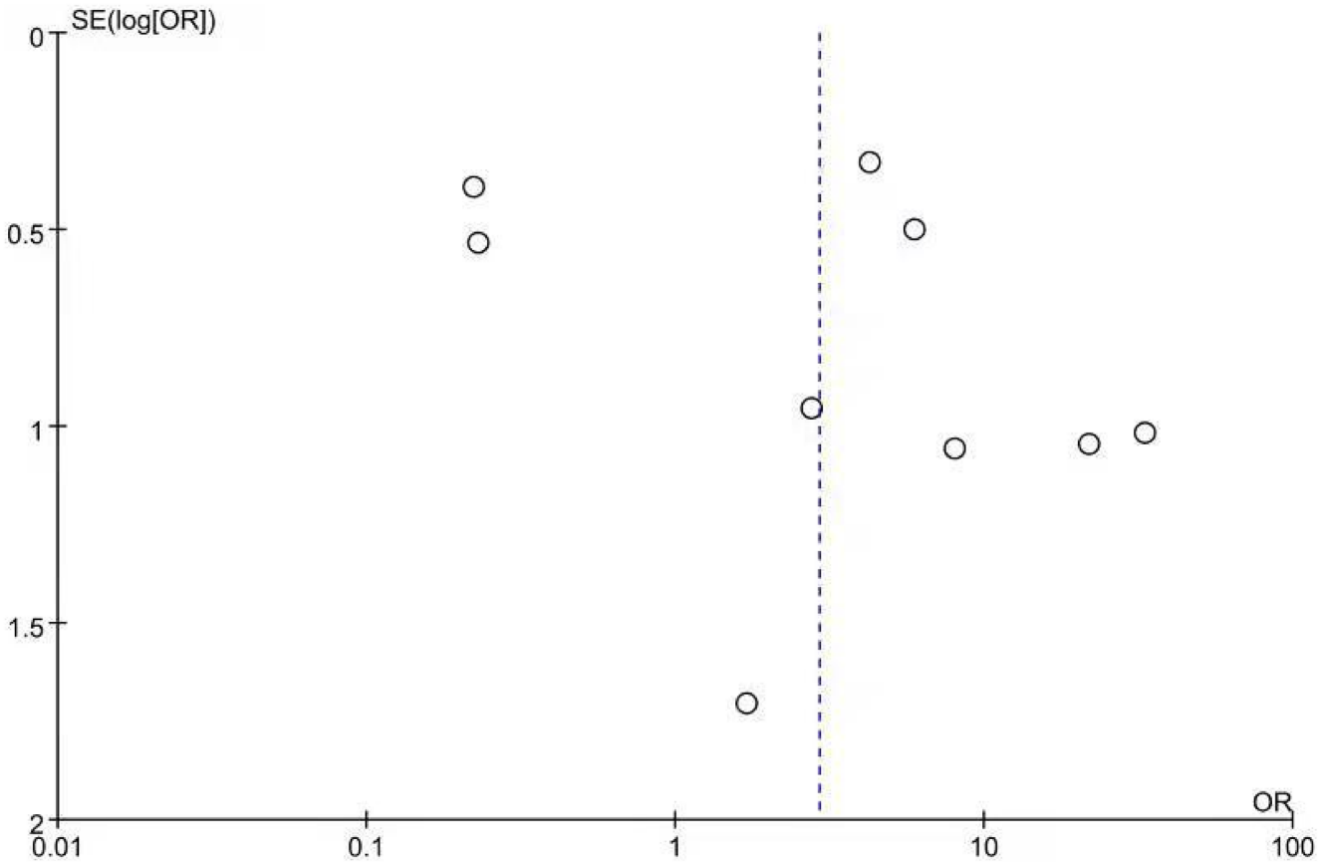
Funnel plot showing the publication bias of odds ratios of SIBO.

**Figure 9:**
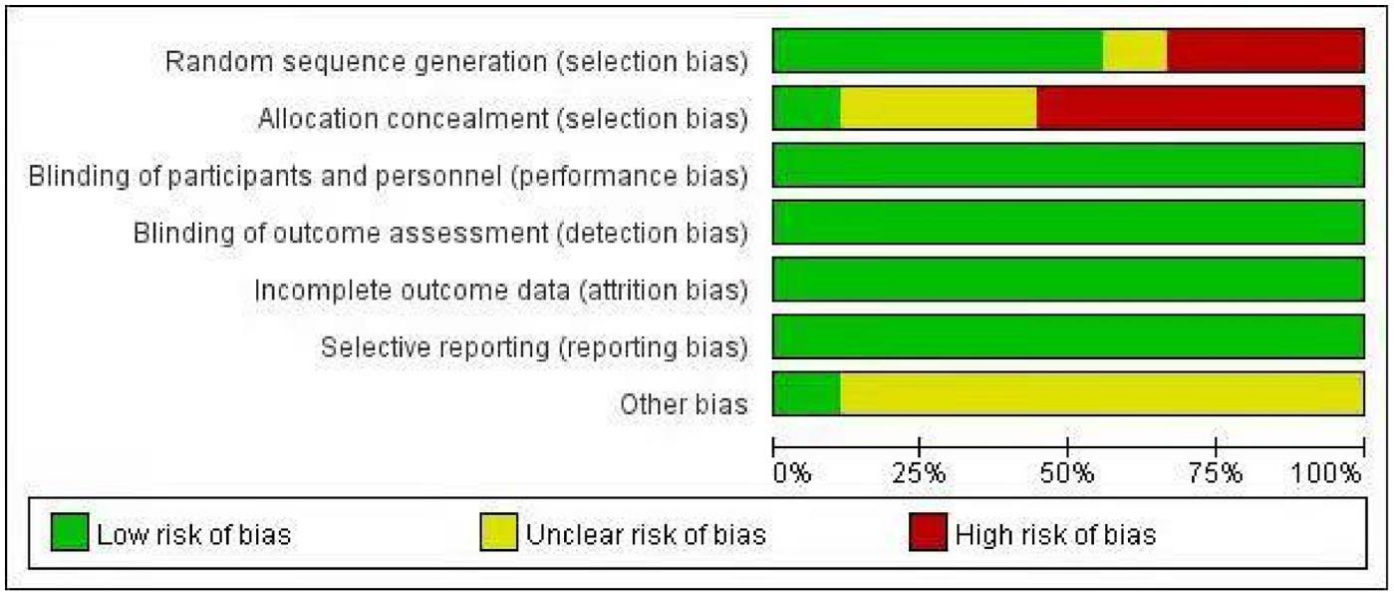
The risk of bias graph about odds ratios of SIBO.

Nine cohort studies [12–14, 19–21, 23, 27, 28] compared the event rate of SIBO between 1105 diabetic patients and 649 controls. The prevalence of SIBO among individuals with DM was higher than that among individuals without DM, with an OR of 2.91 (95% CI 0.82–10.32), although the difference was not statistically significant (p=0.10) (Figure 7). We used random-effects models because of significant heterogeneity (I^2^=89%). The funnel plot indicated a possibility of publication bias (Figure 8). The risk of bias of these studies is graphed in Figure 9.

Due to the significant heterogeneity, a sensitivity analysis was performed by excluding the study by Adamska et al. [13] from the meta-analysis. Exclusion of this study increased the pooled OR to 4.18 (95% CI 1.34-13.05) and reached statistical significance (p=0.01). The between-study heterogeneity was decreased, with an I^2^ of 81%. One reason is that almost half of the controls in the study were recruited from hospital personnel and their relatives, which may have affected the reliability of the results. Another reason is that both studies [13, 14] utilized the same laboratory database of the Poznan University of Medical Sciences during an overlapping period.

## DISCUSSION

To the best of our knowledge, this study is the first systematic review and meta-analysis to investigate the association between DM and the risk of SIBO. Our meta-analysis revealed that (i) the overall pooled prevalence of SIBO in DM was 29% (95% CI 20%–39%), with the variability in SIBO prevalence depending on the type of SIBO diagnostic test and geographic area, and that (ii) the risk of SIBO was 2.91-fold higher among individuals with DM than among individuals without DM.

Mechanisms between DM and SIBO have not been well elucidated. On the one hand, autonomic neuropathy is a common complication in diabetic patients, and it occurs throughout the whole gastrointestinal tract, affecting gastrointestinal motility [29, 30]. Dysfunction of the vagus nerve and intrinsic intestinal autonomic nerves may aggravate gastrointestinal autonomic neuropathy [30]. Gastrointestinal hypomotility due to diabetic autonomic neuropathy can result in small bowel stasis, thereby increasing the likelihood of SIBO. Ojetti et al. [24] found that diabetes patients with autonomic neuropathy have a significantly higher prevalence of SIBO than those without autonomic neuropathy. In addition, oxidative stress and inflammatory cytokines have been demonstrated in previous studies to accelerate the progression of diabetes [31, 32]. Some studies have reported that the levels of inflammatory cytokines (such as IL-6, TNF-α, and IL-10) and oxidative stress-related parameters are significantly higher in both T1DM patients and T2DM patients than in controls [12, 20, 33–40]. In addition, Malik et al. observed that SIBO-positive T2DM patients have a significantly higher level of inflammatory cytokines and oxidative stress than SIBO-negative patients [12]. One explanation is that increased oxidative stress in diabetic patients may result in increased apoptosis of the inhibitory neuronal subpopulation of enteric neurons, which slows gut motility and makes patients more prone to SIBO [41]. On the other hand, SIBO seems to have some impact on diabetic patients. A study by Yan et al. [18] indicated that T2DM patients with SIBO showed worse glycaemic control and a lower level of insulin release than those without SIBO. Similar conclusions were reported in another study in non-alcoholic steatohepatitis (NASH), which indicated that NASH patients with SIBO have a higher prevalence of impaired glucose tolerance than those without SIBO [42]. These results suggest that SIBO could disrupt beta-cell function, although the mechanism remains unclear. One of the hypotheses is that activation of inflammatory pathways reduces insulin secretion by islet cells [43]. Malnutrition and gastrointestinal symptoms are also characteristics of diabetic patients with SIBO. Rana et al. found that urinary d-xylose and lactose intolerance in SIBO-positive T2DM patients was more severe than that in SIBO-negative patients [21]. This indicated that SIBO may aggravate malabsorption and malnutrition and cause various gastrointestinal symptoms. These results were consistent with a study by Yan et al. [18], which showed that T2DM subjects with SIBO had a significantly lower BMI than subjects without SIBO. Malabsorption in SIBO-positive patients might aggravate weight loss [44].

The pooled prevalence of SIBO in patients with DM was 29% in our study. The discrepancies in SIBO prevalence in these studies may be a result of the different SIBO diagnostic tests used and geographic areas. SIBO is a condition in which the small bowel is colonized by excessive numbers of aerobic and anaerobic microbes that are normally found in the large intestine [45, 46]. The gold standard for diagnosing SIBO has long been JAC, although standardized techniques for aseptic collection of intestinal aspirate samples are lacking [45, 47]. The North American Consensus suggests the threshold of >10^3^ colony-forming units per milliliter (c.f.u./ml) for the definition of SIBO [47]. The limitations of JAC are its invasiveness, cost, difficulty accessing the distal small bowel, possible contamination by oral flora, and false negatives for obligate anaerobes [47–49]. Breath tests are non-invasive and inexpensive methods for evaluating SIBO compared to JAC. Carbohydrates are fermented by microbes in the gut to produce gas, which is absorbed into the bloodstream and then expired through the lungs [45, 47]. Breath tests include the LBT and GBT. Lactulose is a non-digestible disaccharide that reaches the colon before a rise in hydrogen or methane and has a higher false-positive result [47, 50–52]. In contrast, glucose is a monosaccharide that is rapidly absorbed in the proximal small bowel, with a higher false-negative result if the bacteria occupy only the lower parts of the small intestine [2, 47, 52, 53]. According to The North American Consensus [47], the correct doses of lactulose and glucose for breath testing are 10 g and 75 g, respectively. A rise of ≥20 parts per million(ppm) above baseline in hydrogen within 90 minutes or a rise of ≥10 ppm in methane should be considered positive for glucose and lactose breath testing, respectively [47]. In our study, the prevalence of SIBO diagnosed by LBT and GBT was lower than that diagnosed by JAC (31% and 29% vs. 39%). This result may be due to contamination with oral and oesophageal flora, resulting in a significant number of false-positive results. The different geographic areas may also account for the variance in reported SIBO prevalence rates in DM. We found that the SIBO prevalence in DM was higher in Western countries than in Eastern countries (35% vs. 24%). One possible explanation for this result is the differences in dietary habits in different countries. High-fat and carbohydrate-rich foods in Western countries can decrease beneficial gut microbes and increase total anaerobic microflora and counts of Bacteroides and Enterobacteriales [54]. Another explanation is the inherently different metabolism and physiology among different ethnic groups. In addition, we observed that the prevalence of SIBO in T1DM was not significantly different from that in T2DM (25% vs. 30%). This suggests that the type of diabetes is not significantly associated with the prevalence of SIBO. Data from the present study suggest that the risk of SIBO is almost three times higher in patients with DM than in controls, although the difference was not statistically significant. Two studies in this meta-analysis reported that the prevalence of SIBO in diabetic patients was lower than that in controls [13, 14], which was not consistent with other studies. One possible reason is that Adamska et al. [13] recruited controls from hospital personnel and their relatives, and all participants in the two studies [13, 14] were from the same medical institution. When we excluded the study by Adamska et al. [13], the risk of SIBO in DM increased to 4.18-fold compared with controls and reached statistical significance.

This study had several limitations: 1) a relatively small sample size due to the limited number of patients in each of the included studies; 2) the result of the funnel plot, which calculates the OR comparing the prevalence of SIBO in DM and controls, suggesting the possibility of publication bias; and 3) different diagnostic tests and different geographic areas of subjects that may have caused heterogeneity in the results.

## CONCLUSIONS

In summary, approximately 29% of diabetic patients tested positive for SIBO. The increased risk of SIBO appears to be greater in patients diagnosed by JAC or in Western populations. There was no significant difference in the prevalence of SIBO between T1DM and T2DM patients. The risk of SIBO in diabetic patients was 2.91 times higher than that in patients without diabetes. These results suggest that DM could be a predisposing factor for the development of SIBO.

## Supplementary Material

Supplementary Table 1
