# Supplementary material for: The prevalence of small intestinal bacterial overgrowth in diabetes mellitus: a systematic review and meta-analysis
Source: Aging (Albany NY). 2022 Jan 27;14(2):975–88. doi: 10.18632/aging.203854 (PMC8833117; doi:10.18632/aging.203854)
Supplement: Supplementary Table 1 [file aging-14-203854-s001.docx]

**Table 1. Main characteristics of the studies included in this meta-analysis.**

| Study | Country | Study design | SIBO diagnos-tic test | SIBO diagnostic criteria | Prevalence of SIBO | type of diabetes | Average age (years) | Gender (male/female) | Course of diabetes (years) | Quality assessment |
| --- | --- | --- | --- | --- | --- | --- | --- | --- | --- | --- |
| Yan et al^[18]^, 2020 | China | cross‐sectional | LBT | 20g lactulose load is orally administered, 1) a baseline H_2_ concentration of >10 ppm 2) H_2_ increase >20 ppm or CH_4_ increase >12 ppm above basal value within 90 minutes. | 56/104 (53.8%) | T2DM | SIBO+:53.52±10.5  SIBO-:53.69±8.39 | Overall 62/42  SIBO+: 34/22  SIBO-: 28/20 | SIBO+:  8.5 (4.25-13.0)  SIBO-:  9.0 (5.00-12.75) | Selection: 4 Comparability: 2 Outcome: 3 |
| Malik et al^[12]^,2020 | India | cohort study | GBT | 70g glucose load is orally administered, an increase of H_2_ ≥12 ppm above the baseline level within 2 hours | cases:43/300(14.3%) controls:1/200 (0.5%) | T2DM | cases: 54.6±0.67 controls:55.4±0.74 | cases:142/158 controls:96/104 | cases:9.6±0.26  controls: none | Selection: 3 Comparability: 2 Outcome: 3 |
| Radionova et al^[19]^,2020 | Ukraine | cohort study | GBT | a rise of H_2_ ≥12 ppm above the basal level after glucose ingestion | cases: 69/92(75%)  controls:33/80(41%) | T2DM | cases: 61.6±9.0 controls:54.0±13.5 | cases: 63/29 controls: 46/34 | not stated | Selection: 3 Comparability: 2 Outcome: 3 |
| Malik et al^[20]^,2018 | India | cohort study | GBT | 80g glucose load is orally administered, rise of H_2_ and/or CH_4_ ≥12 ppm over the baseline value within 2 hours | cases:17/75(22.7%) controls:1/75(1.3%) | T1DM | cases: 22.3 ± 5.2 controls: 23.1±4.9 | cases: 36/39 controls: 37/38 | cases:7.4±3.6 controls: none | Selection: 4 Comparability: 2 Outcome: 3 |
| Rana et al^[21]^,2017 | India | cohort study | GBT | 80g glucose load is orally administered, rise of H_2_ and/or CH_4_ ≥12 ppm over the baseline value within 2 hours | cases:26/175(14.9%) controls:5/175(2.9%) | T2DM | not stated | cases: 87/88 controls: 89/86 | cases:9.6±4.8  controls: none | Selection: 3 Comparability: 2 Outcome: 3 |
| Adamska et al^[13]^,2016 | Poland | cohort study | LBT | 20g lactulose load is orally administered, an elevated fasting H_2_ level >20 ppm or a peak H_2_ level >12 ppm in less than 60 minutes | cases:56/148(37.8%) controls: 30/41(73.2) | T1DM | cases: 45 (35–54) SIBO+: 44 (34–53) SIBO-: 45 (35–54) controls: 31 (27–39) | cases: 94/54 SIBO+: 35/21 SIBO-: 59/33 controls: 17/24 | cases: 20 (13.5–28)  SIBO+: 20 (13-27)  SIBO-: 20 (13.5–29)  controls: none | Selection: 3 Comparability: 2 Outcome: 3 |
| Adamska et al^[14]^,2015 | Poland | cohort study | LBT | 20g lactulose load is orally administered, H_2_ of first breath ≥ 20 ppm or an increase of H_2_ ≥12 ppm within 1 hour | cases: 82/200 (41%) T1DM: 36/91 (39.6%) T2DM:46/109(42.2%) controls: 15/20 (75%) | 91 T1DM  109 T2DM | cases: 54(44–62) controls:37(29–41) | cases: 130/70 controls: 9/11 | cases: 15(10-23) controls: none | Selection:3 Comparability: 2 Outcome: 3 |
| Faria et al^[22]^,2013 | Brazil | cross‐sectional | LBT | 20g lactulose load is orally administered, an early peak (> 10mmHg) detected before 30 min or baseline H_2_ >20 ppm | 3/26 (11.5%) | T1DM | Overall 39±9 | 6/22 | cases: 23±7 | Selection: 3  Comparability: 2 Outcome: 2 |
| Rana et al^[23]^,2011 | India | cohort study | GBT | 80g glucose load is orally administered, an increase of H_2_ and/or CH_4_ ≥12 ppm above the baseline in 2 consecutive readings | cases: 13/84(15.5%) controls: 1/45(2.2%) | T2DM | cases: 40.5±12.6 controls: 43.4±13.8 | cases: 51/33 SIBO+: 6/7 SIBO-: 45/26 controls: 29/16 | cases: 10.5±4.8 controls: none | Selection: 4 Comparability: 2 Outcome: 3 |
| Ojetti et al^[24]^,2009 | Italy | cross‐sectional | LBT | 10g lactulose load is orally administered, a rise of H_2_ >20 ppm by 90 min or 180 min | 13/50(26%) | T1DM | not stated | 28/22 | not stated | Selection: 3 Comparability: 2 Outcome: 2 |
| Urita et al^[25]^,2006 | Japan | cross‐sectional | GBT | 75g glucose load is orally administered, an increase of H_2_ and/or CH_4_ ≥10 ppm above the baseline before the first 40 minutes | 21/82 (25.6%) | not stated | Overall 62 (30–84) | Overall 40/42 | not stated | Selection: 3 Comparability: 2 Outcome: 3 |
| Zietz et al^[26]^,2000 | Germany | cross‐sectional | GBT | 70g glucose load is orally administered, an increase in breath H_2_ concentration >20 ppm | 17/50 (34%) | 20 T1DM  30 T2DM | Overall 47.3±2.2 | not stated | 14.4±1.3 | Selection: 2 Comparability: 2 Outcome: 3 |
| Spengler et al^[27]^,1989 | Germany | cohort study | JAC | ≥10^4^ microorganisms/ml of recovered fluid were considered to indicate increased bacterial growth. | cases: 10/19(52.6%) controls: 2/7(28.6%) | not stated | cases: 40-74 controls: 45-68 | cases: 9/10 controls: 3/4 | not stated | Selection: 2 Comparability: 2 Outcome: 3 |
| Dooley et al^[28]^,1988 | USA | cohort study | LBT/JAC | 10g lactulose load is orally administered, a short (15 min) peak ≥20 ppm of H_2_ before the sustained peak | cases: 1/3/12(25%) controls: 0/6 (0%) | T2DM | cases: 56±8 controls: 36±8 | cases: 2/10 controls: none | cases:13±7 controls: none | Selection: 2 Comparability: 2 Outcome: 3 |
